# EM algorithm for Bayesian estimation of genomic breeding values

**DOI:** 10.1186/1471-2156-11-3

**Published:** 2010-01-22

**Authors:** Takeshi Hayashi, Hiroyoshi Iwata

**Affiliations:** 1 Division of Animal Sciences, National Institute of Agrobiological Sciences, Kannondai, Tsukuba, Ibaraki 305-8602, Japan; 2 Data Mining and Grid Research Team, National Agricultural Research Center, Kannondai, Tsukuba, Ibaraki 305-8666, Japan

## Abstract

**Background:**

In genomic selection, a model for prediction of genome-wide breeding value (GBV) is constructed by estimating a large number of SNP effects that are included in a model. Two Bayesian methods based on MCMC algorithm, Bayesian shrinkage regression (BSR) method and stochastic search variable selection (SSVS) method, (which are called BayesA and BayesB, respectively, in some literatures), have been so far proposed for the estimation of SNP effects. However, much computational burden is imposed on the MCMC-based Bayesian methods. A method with both high computing efficiency and prediction accuracy is desired to be developed for practical use of genomic selection.

**Results:**

EM algorithm applicable for BSR is described. Subsequently, we propose a new EM-based Bayesian method, called wBSR (weighted BSR), which is a modification of BSR incorporating a weight for each SNP according to the strength of its association to a trait. Simulation experiments show that the computational time is much reduced with wBSR based on EM algorithm and the accuracy in predicting GBV is improved by wBSR in comparison with BSR based on MCMC algorithm. However, the accuracy of predicted GBV with wBSR is inferior to that with SSVS based on MCMC algorithm which is currently considered to be a method of choice for genomic selection.

**Conclusions:**

EM-based wBSR method proposed in this study is much advantageous over MCMC-based Bayesian methods in computational time and can predict GBV more accurately than MCMC-based BSR. Therefore, wBSR is considered a practical method for genomic selection with a large number of SNP markers.

## Background

Genome-wide polymorphisms are increasingly elucidated in livestock and crops with the recent development of the sequencing technologies. Accordingly, high-throughput genotyping systems, such as high-density SNP chips containing several tens of thousands of genome-wide SNP markers, have become available to efficiently identify genotypes of individuals for a large number of SNPs with low cost. As a new breeding technology utilizing the information of genome-wide dense SNP markers, genomic selection was proposed by Meuwissen et al. (2001) [1]. In genomic selection, firstly a well-fitted model for genomic breeding value (GBV) of a trait is constructed by estimating SNP effects included in the model as parameters using the individuals with data of both genotypes of SNPs and phenotypes of a trait (training data set). Secondly, GBV is predicted for individuals to be selected based on only genotype data of SNPs (selection candidates) using the fitted model. For the estimation of SNP effects, two Bayesian methods called BayesA and BayesB were proposed as well as a BLUP method and it was shown that BayesB could predict GBV most accurately of the methods using simulation experiments [1].

BayesA method can be classified into a method of Bayesian shrinkage regression (BSR) [2] from a view point of statistical methodology, which can handle a large number of model effects requiring no variable selection. In BSR, a model including of effects of all SNPs available are considered and the shrinkage estimation is applied for these SNP effects assuming the appropriate prior distribution for the effects such as a normal distribution with a mean 0. On the other hand, BayesB method can be regarded as a modified version of stochastic search variable selection (SSVS) [3]. In the original SSVS method, each SNP effect (regression coefficient) is assigned a mixture of two normal distributions both having means 0 but one with a large variance and the other with a tiny variance. If the posterior probability of the effect to belong to the distribution with a large variance is high, this effect is considered as selected and included in the model. In the method of BayesB, a mixture of a normal distribution with a mean 0 and a large variance and a distribution with point mass only at zero which might be regarded as a normal distribution with both of a mean and a variance set at zero is assumed for each SNP effect. Meuwissen et al. [1] used block-updating for a SNP effect and a variance to prevent the estimate from being stuck at zero. In this simultaneous update, a variance is assigned a zero or sampled from a prior inverted chi-square distribution following a prior mixture probability, which is a prior probability of each SNP to be included in the model, and then a SNP effect is obtained from a conditional normal distribution given a variance. Taking these things into consideration, we use more general statistical terms BSR and SSVS for BayesA and BayesB, respectively, hereafter in this paper for the help of understanding of readers in broad research fields. Although BayesB can be interpreted as a variant of original SSVS as noted above, we use the term 'SSVS' for BayesB, which could cause no confusion.

Usually, Markov chain Monte Carlo (MCMC) algorithm has been applied to the model construction with BSR and SSVS in genomic selection. However, MCMC-based Bayesian methods are much time-consuming and therefore might be prohibited for application as the sample size and/or the number of SNPs become much larger. Accordingly, a fast non-MCMC algorithm for SSVS utilizing the analytical form of posterior means of SNP effects was devised [4], where conditional posterior expectation of each SNP effect could be analytically calculated by assuming a mixture of a distribution with a discrete probability mass of zero and a double exponential distribution for a prior distribution for SNP effects. It was shown that this analytical SSVS method was slightly inferior to MCMC-based SSVS but much superior to BLUP in the accuracy of predicting GBV. It was also shown that this analytical SSVS predicted GBV in a very similar way as MCMC-based one with much reduced computing time [4].

Xu (2003) [2] proposed BSR in the context of mapping QTL effects on a whole genome to capture the polygenic effects. This shrinkage mapping method was improved and extended by some authors [5-7]. The efficiency of QTL mapping using BSR was shown to be superior to that using SSVS in [5]. Recently, Yi and Benerjee (2009) [8] proposed an EM-based algorithm for the maximization of the posterior distribution function in BSR.

In this study, we apply the EM algorithm described in [8] for the model construction including estimation of SNP effects in BSR from a view point of genomic selection. Although generalized linear models were considered to deal with several types of phenotypes including categorical traits and continuous polygenic traits in [8], we confine ourselves to the case of continuous traits here for simplicity. Moreover, we incorporate the weight for each SNP according to the strength of its association with a trait in the procedure of model construction with BSR to improve the prediction accuracy. The weight of SNP can be regarded as an approximate posterior probability of SNP to be included in a model and obtained from a given prior probability of SNP inclusion with EM algorithm. We call this model construction procedure as wBSR, which means a modified BSR incorporating the weights for SNPs.

Using the simulation experiments, we compare the accuracies of EM-based wBSR with BSR and SSVS using MCMC algorithm in the prediction of GBV for several values of the prior probability, *p*, of SNP inclusion in the model. It is shown that the accuracy of wBSR can be improved in comparison with MCMC-based BSR although the accuracy of wBSR is inferior to SSVS and is influenced by the values of *p *and the hyperparameters of the prior inverted chi-square distribution assumed for the variances of SNP effects. Moreover, the computational cost of wBSR is much less than the MCMC-based Bayesian methods. Therefore, wBSR is considered a practical and useful method for genomic selection with a large number of SNP markers.

## Methods

In this section, we will describe the methods of BSR (BayesA) and SSVS (BayesB) for genomic selection and EM algorithm for BSR to obtain the estimates of parameters included in the model that maximize the posterior distribution function. Subsequently, we will modify BSR method (wBSR) by assigning the weight for each SNP according to the strength of its association to a trait for improvement of the prediction accuracy. The weight of SNP can be obtained from a prior probability of each SNP to be included in a model, which is also considered in SSVS procedure, using EM algorithm as well as the estimate of SNP effect. For the evaluation of the accuracies of the predicted GBVs, we apply wBSR with variable prior probabilities of SNP inclusion for simulated data sets as well as MCMC-based BSR and SSVS.

In the statistical model described below, we consider not haplotype effect but the effect of each single SNP. We assume that the number of SNPs genotyped is *N *and a training data set including *n *individuals with the records of phenotypes and SNP genotypes is available for the estimation of parameters in the model. We also assume that selection candidates consists of individuals with only SNP genotypes, for each of which GBV is predicted based on the model with SNP effects estimated with training data sets. We denote two alleles at each SNP by 0 and 1 and three genotypes by '0_0', '0_1', and '1_1'.

### Models for BSR and SSVS in genomic selection

In BSR (BayesA) method [1,2], the following linear model is fitted to the phenotypes of a training data set:(1)

where **y **= (*y*_1_, *y*_2_, ..., *y*_*n*_)' is a vector of phenotypic values of a trait for *n *individuals of a training data set, **u**_*l *_= (*u*_*l*1_, *u*_*l*2_, ..., *u*_*ln*_)' is a vector of genotypes of *n *individuals at the *l*th SNP with *u*_*li *_taking a value of -1, 0, or 1 corresponding to the genotypes '0_0', '0_1', or '1_1', respectively, *g*_*l *_is the effect of the *l*th SNP, **b **= (*b*_1_, *b*_2_, ..., *b*_*f*_)' is a vector of fixed non-genetic effects with dimension *f *including a general mean, **X **= (*x*_*ij*_) (*i *= 1, 2, ..., *n*; *j *= 1, 2, ..., *f*) is a design matrix relating **b **to **y **and **e **= (*e*_1_, *e*_2_, ..., *e*_*n*_)' is a vector of random deviates with *e*_*i *_~*N*(0, *σ*_*e*_^2^). It is assumed that the prior distribution of the SNP effect, *g*_*l*_, is a normal distribution with a mean 0 and a variance *σ*_*gl*_^2^, which differs for every SNP. Moreover, the prior distribution of *σ*_*gl*_^2 ^is considered. In this study, we assume that it is a scaled inverted chi-squared distribution with a scale parameter *S *and a degree-of-freedom *ν*, *χ*^-2^(*ν*, *S*), following [1,2]. The posterior distributions of relevant parameters, **b**, *g*_*l*_, *σ*_*gl*_^2 ^(*l *= 1, 2, ..., *N*) and *σ*_*e*_^2^, can be obtained by Gibbs sampling [1,2]. For the individuals of selection candidates, GBV are predicted by , where  is the estimate of *g*_*l*_. In this study, we consider not haplotype effect but the single marker effect for *g*_*l*_. The use of marker haplotypes instead of the single marker genotypes would cause slight modification of the model, but the procedure for estimation of effects and prediction of GBV is essentially the same.

In SSVS (BayesB) method, the model (1) is also adopted but a prior probability, *p*, of each SNP to be included in the model is considered. Usually, a small value is given for *p *based on the assumption that many of SNPs have actually no effects for a trait. The prior distribution of *g*_*l *_is assumed to be a normal distribution with a mean 0 and a variance *σ*_*gl*_^2 ^in SSVS as in BSR, whereas the prior distribution of *σ*_*gl*_^2 ^is expressed as a mixture of two distributions corresponding to the inclusion and the exclusion of the SNP as follows:

assuming that the prior is *χ*^-2^(*ν*, *S*) when the SNP is included. When MCMC algorithm is applied for the estimation of the parameters in SSVS, *g*_*l *_and *σ*_*gl*_^2 ^are jointly updated with Metropolis-Hastings chain [1]. The GBV predicted by SSVS is presented by  as in BSR.

### EM algorithm for BSR

In Bayesian estimation, the inferences about the parameters are made based on the posterior distributions. MCMC algorithms can be used for obtaining the posterior information of the parameters in BSR method as described above. However, the posterior mode of each SNP effect which is a point estimate maximizing the density function of the posterior distribution can be calculated instead of a posterior expectation by some other iteration algorithm including EM algorithm. In QTL mapping using BSR method, Yi and Banerjee [8] utilized an EM algorithm to search the posterior mode of the marker effects included in the model. This EM algorithm can be applied for genomic selection with BSR method without any modification and we describe the estimation procedure for the EM algorithm in this section. Although, in [8], phenotypic data was transformed to have a mean 0 and a standard deviation 0.5 following Gelman et al. (2008) [9] and the derivations of the posterior estimates of parameters were illustrated in the framework of generalized linear model, original phenotypic data are subject to the EM algorithm here without any transformation and we derive the posterior estimates of parameters under the normality in what follows assuming that the trait of concern is polygenic and normally distributed.

The posterior distribution is given by combining a likelihood of the data and the prior distributions of the parameters. We denote parameters in BSR method as a vector form **θ**,

The posterior distribution of **θ **given the data of phenotypes, **y**, and genotypes of SNP data, **U **= (**u**_1_, **u**_2_, ..., **u**_*N*_), is denoted by *g*(**θ **| **y**, **U**) and written as(2)

where *C *means a constant and it should be noted that the likelihood of **y **given the model parameters and genotypes is a normal distribution with a mean **Xb **+  and a variance *σ*_*e*_^2 ^and the prior of *g*_*l *_is a normal distribution with a mean 0 and a variance *σ*_*gl*_^2^, the prior of which is the scaled inverted chi-squared distribution *χ*^-2^(*ν*, *S*) as described above. The priors of **b **and *σ*_*e*_^2 ^are written by *p*(**b**) and *p*(*σ*_*e*_^2^), respectively, which are assumed uniform distributions over suitable ranges of the values here.

Following [8], we regard the variances of SNP effects, *σ*_*gl*_^2 ^(*l *= 1, 2, ..., *N*), as missing data and replace *σ*_*gl*_^2 ^by the conditional posterior expectation of *σ*_*gl*_^2^, denoted by , given other parameters as E-step in the EM algorithm. Considering the expectation of scaled inverted chi-square distribution, it is given as(3)

As M-step, we obtain the values of parameters other than *σ*_*gl*_^2 ^(l = 1, 2, ..., *N*) maximizing the log-posterior distribution with *σ*_*gl*_^2 ^replaced by , which is expressed from (3) as

log *g*(**θ**|**y**, **U**)

The mode of each parameter which maximizes the log-posterior can be given by solving an equation derived by making the partial derivative of the log-posterior with respect to the parameter equal to 0. Accordingly the modes of *g*_*l*_(*l *= 1, 2, ..., *N*), *b*_*j *_(*j *= 1, 2, ..., *f*) and *σ*_*e*_^2^, denoted as ,  and , satisfy the following equations:(4)

The EM algorithm for BSR is summarized as follows:

1. E-step: *σ*_*gl*_^2 ^is estimated as  shown in (3) that is a conditional expectation given a current value of *g*_*l*_, which is , for *l *= 1, 2, ..., *N*.

2. M-step: the values of *g*_*l *_(*l *= 1, 2, ..., *N*), *b*_*j *_(j = 1, 2, ..., *f*) and *σ*_*e*_^2 ^maximizing the log posterior distribution of parameters, ,  and , are given according to (4), (5) and (6), where the value of each parameter are updated by replacing the other parameters by their current values.

E-step and M-step are repeated until the values of parameters converge. We stop this iteration when the change of values of parameters becomes small. For example, when  < 10^-6^, where  and  are the current and the previous value of the parameters, the EM algorithm is regarded to be converged. We adopt this criterion for convergence of EM algorithm in the study.

### Modification of BSR

In SSVS, SNP effects can shrink more strongly than in BSR due to the assumption that only a small number of SNPs can be linked to QTL causing only a small portion of SNPs to have significant effects and many other SNPs to have negligible effects, which might result in the improvement of prediction accuracy for SSVS using a more parsimonious model. Although it was reported in [5] that BSR could provide a more accurate result for QTL mapping with less than a hundred markers than SSVS developed by Yi et al. (2003) [10], SSVS that is capable of deleting many SNPs with ignorable effects might perform as well or better than BSR in the case of a huge number of high-density SNPs involved in the prediction of GBV. However, the EM algorithm described above cannot be applied to SSVS because the prior distribution of *σ*_*gl*_^2^, a mixture distribution combining *χ*^-2^(*ν*, *S*) and 0 with probability *p *and 1-*p*, respectively, cannot be well treated with EM algorithm. To devise a cost-effective and EM-based method providing more accurate prediction for genomic selection with a higher degree of shrinkage, we develop a new modified BSR method incorporating a weight for each SNP depending on the strength of its association with a trait. In this method, we modify the model (1) by incorporating the variable *γ*_*l *_indicating the inclusion of the *l*th SNP in the model or exclusion of the *l*th SNP from the model, where inclusion and exclusion of the SNP are indicated by *γ*_*l *_= 1 and *γ*_*l *_= 0, respectively. We assume that the prior probabilities of *γ*_*l *_= 1 and *γ*_*l *_= 0 are *p *and 1-*p*, respectively, as in SSVS. The modified model is written as(7)

where **X**, **b**, **u**_*l*_, *g*_*l *_and **e **are as described in the model (1). We assume that the priors of *g*_*l *_and *σ*_*gl*_^2 ^are not influenced by the inclusion (*γ*_*l *_= 1) or exclusion (*γ*_*l *_= 0) of SNP in the model (2) and are as adopted in BSR. The method with the model (7), but utilizing these assumption, is called wBSR, meaning a modified BSR incorporating SNP weight, in this study since the same EM procedure as used in BSR for searching the posterior mode of parameters can be applied for this method and it is equivalent to an EM-based BSR procedure proposed by [8] when *p *= 1. We denote the variables indicating the inclusion of SNP effects in the model in a vector form as **γ **= (*γ*_1_, *γ*_2_, ..., *γ*_*N*_) which are treated as variables to be estimated in wBSR.

In wBSR, the posterior distribution *g*(**θ**, **γ **| **y**, **U**) is modified from (2) and written as *g*(**θ**, **γ **| **y**, **U**)(8)

where the priors *p*(**b**) and p(*σ*_*e*_^2^) are assumed uniform distributions. Applying the same argument as in EM algorithm used for BSR, *σ*_*gl*_^2 ^is replaced by its conditional posterior expectation, , in E-step which is given in (3). The variable *σ*_*l *_indicating the inclusion of SNP in the model is unobserved, thus, *σ*_*l *_is also replaced by its conditional posterior expectation *ξ*_*l *_which can be written, from (8) and under the assumption that the priors of *g*_*l *_and *σ*_*gl*_^2 ^are independent of *σ*_*l*_, as

where **γ**_-*l *_denotes **γ **with the *l*th component *γ*_*l *_deleted and

In this expression, however, *γ*_*j *_(*j *≠ *l*) is also unobserved. Therefore, we modify the expression for *ξ*_*l *_by substituting *γ*_*j *_with *ξ*_*j *_for *j *≠ *l*. Accordingly, the conditional posterior expectation of *γ*_*l*_, *ξ*_*l*_, is approximately obtained in E-step for *l *= 1, 2, ..., *N *following the formula:(9)

where 

In M-step, the values of *b*_*j *_(*j *= 1, 2, ..., *f*) and *σ*_*e*_^2 ^maximizing g(**θ**| **y**, **U**), , and , satisfy the equations that are slightly changed from (6) and (7) and given as

and

For *g*_*l*_, the value maximizing the posterior (8), , depends on *γ*_*l *_and is given as  = 0 for *γ*_*l *_= 0 and

for *γ*_*l *_= 1. As *γ*_*l *_is unobserved, we substitute *ξ*_*l *_for *γ*_*l *_in the expressions of ,  and . For , the expression corresponding to *γ*_*l *_= 1 is adopted for the iteration. In summary, ,  and  calculated in M-step are given as(10)

and(12)

It should be noted that *ξ*_*l *_given by (9) is an approximate posterior expectation of *γ*_*l *_that might be different from the posterior probability of SNP to be included in the model. Therefore, *ξ*_*l *_is referred to as the weight of the SNP that is regarded as an indicator of the strength of the association of the SNP with a trait. The SNP assigned a large weight with *ξ*_*l *_taking values near one is considered to essentially contribute to GBV while the contribution of the SNP assigned a small weight with *ξ*_*l *_taking values near the given prior value of *p *is regarded as negligible. The degree of shrinkage can be affected by the value of a prior probability *p *as well as the values of hyperparameters, *ν *and *S*, in *ξ*^-2^(*ν*, *S*), the prior distribution for *σ*_*gl*_^2^. The predicted GBV of wBSR is expressed as 

### Simulation experiments

We evaluated the accuracy for the prediction of GBV using wBSR with variable *p *based on simulated data sets. The population and genome were simulated following the way as in [11]. In brief, the populations with an effective population size 100 were maintained by random mating for 1000 generations to attain mutation drift balance and linkage disequilibrium between SNPs and QTLs. The genome was assumed to consist of 10 chromosomes with each length 100 cM. Two scenarios were considered for the number of SNP markers available in the simulations and data sets under two scenarios were denoted as Data I and Data II. In Data I, 101 marker loci were located every 1 cM on each chromosome with total of 1010 markers on a genome. In Data II, 1010 equidistant marker loci were located on each chromosome with a total of 10100 markers. We assumed that equidistant 100 QTLs were located on each chromosome such that a QTL was in the middle of every marker bracket in Data I and the middle of every 10th marker bracket in Data II. Therefore, there were a total of 1000 QTLs located on a whole genome. The mutation rates assumed per locus per meiosis were 2.5 × 10^-3 ^and 2.5 × 10^-5 ^for marker locus and QTL, respectively. At least one mutation occurred in the most of all marker loci with such high mutation rate during the simulated generations. In the marker loci experiencing more than one mutation, the mutation remaining at the highest minor allele frequency (MAF) was regarded as visible, whereas the others were ignored, which caused the marker loci to have two alleles like SNP markers. The polymorphic QTLs at which mutation occurred only affected the trait, where the effects of QTL alleles were sampled from a gamma distribution with scale parameter 0.4 and shape parameter 1.66 and were assigned with positive or negative values with equal probabilities [1,11].

In generation 1001 and 1002, the population size was increased to 1000. The population in the 1001th generation was treated as a training data, where the phenotypes of a trait and SNP genotypes of the individuals were simulated and analyzed with methods of genomic selection to estimate the SNP effects in the model. The phenotype of each individual in the 1001th generation was given as a sum of QTL effects over the polymorphic QTLs and environmental effects (or residuals) sampled from a normal distribution with a mean 0 and a variance 1 such that the heritability in the population was expected to be 0.5. The population in the 1002th generation was used as selection candidates, where the individuals were only genotyped for 1010 and 10100 SNP markers in Data I and Data II, respectively, without phenotypic records and GBV of each individual was predicted using a model with SNP effects estimated based on the population in the 1001th generation. The true breeding value (TBV) of the individual in the 1002th generation was also simulated as a sum of QTL effects corresponding to the QTL genotype and utilized for evaluation of the accuracy of predicted GBV but was regarded as unknown and unavailable in the estimation of SNP effects in the models. The accuracy was measured by the correlation between the predicted GBV and TBV.

For the evaluation of the accuracies of the predicted GBVs obtained by wBSR with *p *= 0.01, 0.05, 0.1, 0.2, 0.5 and 1.0, we simulated 100 and 20 data sets under the scenario of Data I and Data II, respectively. The accuracies of the GBVs predicted by BSR and SSVS based on MCMC algorithm were also evaluated on the same data sets in comparison with wBSR. In MCMC iteration, we repeated 11000 cycles using a burn-in period of the first 1000 cycles. The values of parameters were sampled every 10 cycles for obtaining the posterior means. In SSVS, we investigated the accuracies of predicted GBVs for *p *= 0.01, 0.05, 0.1, 0.2 and 0.5 in Data I but for *p *= 0.01, 0.05 and 0.1 in Data II due to large computational time required for MCMC algorithm. SNP markers with MAF less than 0.05, which were less than 10% of all SNPs, were not used for the estimation of effects and the prediction of GBV. We set *ν *= 4.012 and *S *= 0.002 for MCMC-based BSR and wBSR with *p *= 1.0 that is equivalent to an EM-based BSR proposed by [8], and *ν *= 4.234 and *S *= 0.0429 for SSVS and wBSR with other values of *p*. These values of *ν *and *S *were determined following [1].

## Results

The accuracies of the predicted GBVs obtained by several methods for genomic selection were evaluated in 100 simulated data sets of Data I and in 20 data sets of Data II, where we assumed that 1010 SNP markers and 10100 SNP markers were available on a whole genome for Data I and Data II, respectively. The results of the simulations were summarized in Table 1, where the regression coefficients of the true GBV on the predicted GBV were also listed for the purpose of reference as well as the correlation coefficients. Although we evaluated the accuracies of the prediction of GBV with the correlation coefficients, the regression coefficient could be used as an indicator of bias for the predicted GBV.

**Table 1:** Accuracies of prediction of GEV in the methods of genomic selection

Methods		Data I	Data II
wBSR	*p *= 0.01	0.699 ± 0.007 (0.950 ± 0.005)	0.843 ± 0.014 (0.961 ± 0.009)
	*p *= 0.05	0.730 ± 0.006 (0.947 ± 0.005)	0.857 ± 0.012 (0.871 ± 0.010)
	*p *= 0.1	0.743 ± 0.006 (0.940 ± 0.005)	0.848 ± 0.014 (0.882 ± 0.016)
	*p *= 0.2	0.755 ± 0.006 (0.924 ± 0.005)	0.820 ± 0.017 (0.795 ± 0.022)
	*p *= 0.5	0.760 ± 0.005 (0.868 ± 0.007)	0.665 ± 0.023 (0.507 ± 0.031)
	*p *= 1.0	0.697 ± 0.007 (1.080 ± 0.008)	0.840 ± 0.015 (0.914 ± 0.017)
BSR		0.748 ± 0.006 (1.100 ± 0.007)	0.838 ± 0.015 (0.885 ± 0.019)
SSVS	*p *= 0.01	0.718 ± 0.007 (1.033 ± 0.006)	0.887 ± 0.011 (1.002 ± 0.009)
	*p *= 0.05	0.747 ± 0.006 (1.036 ± 0.005)	0.874 ± 0.012 (0.942 ± 0.013)
	*p *= 0.1	0.762 ± 0.005 (1.027 ± 0.005)	0.846 ± 0.014 (0.865 ± 0.018)
	*p *= 0.2	0.772 ± 0.005 (1.008 ± 0.005)	n.d.
	*p *= 0.5	0.773 ± 0.005 (0.944 ± 0.005)	n.d.

The means of correlation coefficients between the predicted GBV over 100 and 20 repetitions in Data I and Data II, respectively, are listed with the standard errors. The means of regression coefficients of true on predicted GEV are given with the standard errors in the parenthesis.

wBSR: EM-based modified BSR method proposed in this paper.

BSR: MCMC-based Bayesian shrinkage regression method (BayesA).

SSVS: MCMC-based stochastic search variable selection method (BayesB).

For the parameters *ν *and *S*, we set *ν *= 4.012 and *S *= 0.002 for BSR and wBSR with *p *= 1.0 (EM-based BSR) and *ν *= 4.234 and *S *= 0.0429 for SSVS and wBSR with *p *< 1.0.

"n.d." indicates that the analysis was not done.

In Data I, SSVS based on MCMC-algorithm provided the most accurate prediction for GBV with the accuracy of 0.772 when *p *= 0.5 in the given settings of *ν *and *S *(Table 1). The accuracy of wBSR was affected by the value of *p *and reduced as the value of *p *was decreased from 0.5. The accuracies of wBSR was 0.760 at *p *= 0.5 and reduced to 0.699 at *p *= 0.01 in the same setting of *ν *and *S*. This was the case for SSVS, where the accuracy of SSVS ranged from 0.772 at *p *= 0.5 to 0.718 at *p *= 0.01. The prediction accuracies with MCMC-based BSR and EM-based BSR (wBSR with *p *= 1.0) were considerably different in Data I. MCMC-based BSR provided significantly better predicted GBV with accuracy of 0.748 than EM-based BSR with accuracy of 0.697 considering the standard errors based on 100 repetitions as shown in Table 1. It was shown that the accuracy was significantly improved with wBSR at *p *= 0.5 in comparison with MCMC-based BSR in Data I although different values of *ν *and *S *were assumed. In Data II, SSVS with *p *= 0.01 could predict GBV most accurately with the accuracy of 0.887. The accuracy of wBSR was influenced by the value of *p *also in Data II, which was 0.843 at *p *= 0.01 and attained to 0.857 at *p *= 0.05 but much reduced to 0.665 at *p *= 0.5 (Table 1). The accuracy of SSVS was reduced to 0.874 and 0.846 with *p *= 0.05 and *p *= 0.1, respectively. MCMC-based and EM-based BSR provided similar accuracies in Data II, which were 0.838 and 0.840, respectively.

**Figure 1:**
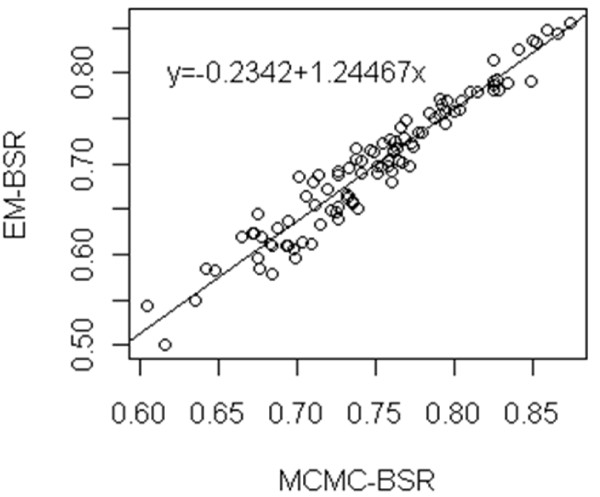
**Plot of the prediction accuracy for GBV with MCMC-based BSR against that with EM-based BSR in 100 repetitions of Data I**.

**Figure 2:**
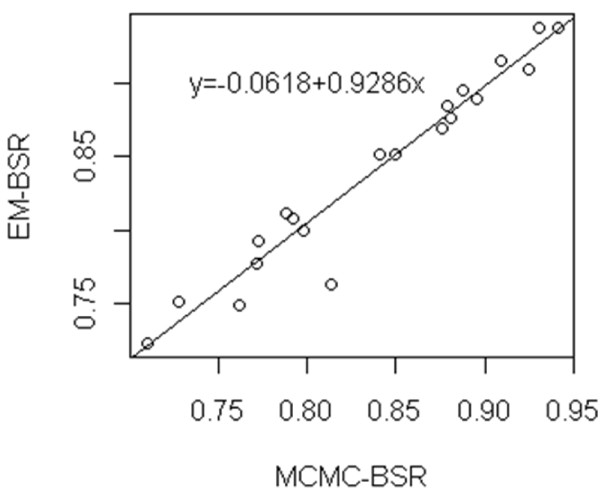
**Plot of the prediction accuracy for GBV with MCMC-based BSR against that with EM-based BSR in 20 repetitions of Data II**.

In EM-algorithm used for wBSR, the posterior modes of SNP effects maximizing the posterior distribution are obtained whereas the posterior expectations of SNP effects are given using MCMC estimation. Therefore, some inconsistency might be anticipated for the estimates of SNP effects, which might make the difference between accuracies of GBVs predicted by MCMC-based BSR and its EM-based version, wBSR with *p *= 1.0. In Data I, the difference between the accuracies with MCMC-based and EM-based BSR was significant as shown in Table 1. In Data II, however, the accuracies with both types of BSR well agreed. We plotted the accuracy obtained by MCMC-based BSR in the analysis of each data set against that by EM-based BSR for Data I and Data II in Figure 1 and Figure 2, respectively. As seen in Figure 1, the inconsistency between the accuracies with MCMC-based BSR and that with EM-based BSR appeared to be small in Data I although they were significantly deviated from each other. The good consistency of the accuracies with both ESR methods was visible in Data II as shown in Figure 2. However, goodness of the agreement between MCMC-based and EM-based BSR seemed dependent on the property of analyzed data.

## Discussion

In this study, EM algorithm for the estimation of SNP effects in BSR method for genomic selection was described following the algorithm proposed in QTL mapping [8]. Moreover, BSR method was modified by incorporating the weight assigned to each SNP in the model reflecting the strength of its association with a trait for controlling the degree of shrinkage. For this method of wBSR, the EM algorithm could be also applied. The computational advantage of the wBSR method over MCMC-based Bayesian methods was obvious and would become remarkable as the number of SNP markers increased. In the simulations, wBSR took less than 30 seconds for the estimation of all SNP effects in each data set of Data I (1010 SNPs) and less than 2 minutes in each data set of Data II (10100 SNPs) on the average, whereas MCMC-based SSVS took more than 30 minutes and more than four hours in each data set of Data I and Data II, respectively, when *p *= 0.05 on the average using a dual processor 2 GHz machine (Intel Xeon 2 GHz) without parallel computing implementation. Although the computational time required by MCMC-based BSR was less than that by SSVS, it still took more than 25 minutes and more than three hours on average in the analysis of a single data set of Data I and Data II, respectively. The iteration times in wBSR until attaining to convergence based on the criterion adopted here ranged 30 to 120 depending on the simulated data.

A fast non-MCMC algorithm for SSVS method, called fBayesB, was proposed in [2]. In this method, the posterior expectation of each SNP effect, *g*_*l*_, was analytically evaluated instead of MCMC-based numerical calculation, where the prior of *g*_*l *_was assumed to be a mixture of a distribution with a discrete probability mass of zero and a double exponential distribution. Although no comparison between this method of SSVS based on the analytical integration and wBSR proposed here was made in this study, the simulation experiments showed that wBSR was also effective in computational time based on EM algorithm, which is a simple algorithm without integral calculation, and performed better than MCMC-based BSR, thus, wBSR could be regarded as a simpler method for genomic selection with practical prediction accuracy and computing efficiency as well as the SSVS method utilizing analytical integration (fBayesB).

As shown in Table 1, the accuracy of GBV predicted was much influenced by the value *p*, a prior probability of SNP to be included in the model. The accuracy was considered to also change along with the values of hyperparameters, *ν *and *S*, in *χ*^-2^(*ν*, *S*), the prior distribution for *σ*_*gl*_^2^. These prior parameters given a priori determine the degree of shrinkage of estimation for SNP effects and affect the accuracy of the prediction of GBV as well as the property of data analyzed. We adopted here the values of *ν *= 4.234 and *S *= 0.0429 for SSVS and wBSR with *p *< 1.0 and *ν *= 4.012 and *S *= 0.002 for MCMC-based BSR and EM-based BSR (wBSR with *p *= 1.0) since we considered the same scenario in simulations as that used by [1] for the population size, mutation rates of markers and QTL and the number of QTL, in which these values of *ν *and *S *were theoretically calculated as suitable values for SSVS and BSR. However, the suitability of these values of *ν *and *S *might be affected by the structure of analyzed data such as the number of SNPs involved, especially for BSR including all of SNPs in the model. Therefore, we performed additional analyses with MCMC-based and EM-based BSR for Data I and Data II using the different values of *ν *and *S*. We adopted the same setting of *ν *and *S *as used in SSVS (that is, *ν *= 4.234 and *S *= 0.0429), which should cause less shrinkage for the estimate of SNP effect, in the additional analysis with both types of BSR in Data I. In Data II, the Jeffreys' prior *p*(*σ*_*gl*_^2^) ∝ 1/*σ*_*gl*_^2 ^corresponding to *ν *= 0.0, yielding strong shrinkage for very small SNP effect but weak shrinkage for large effects [8], was tested for the analysis with both types of BSR. In the additional analysis of 100 simulated data sets in Data I with the same setting of *ν *and *S *as in SSVS, the accuracy of EM-based BSR (wBSR with *p *= 1.0) much increased from 0.697 to 0.744 with standard error (s.e.) of 0.006 while the increase in the accuracy of MCMC-based BSR was slight, where the accuracy was changed from 0.748 to 0.754 with s.e. of 0.006. In another additional analysis of 20 repetitions of Data II using the Jeffreys' prior, the accuracies of both types of BSR were decreased in comparison with the original prior setting of *ν *and *S*. We obtained the accuracy of 0.834 with s.e. 0.017 for MCMC-based BSR and the accuracy of 0.809 with s.e. 0.016 for EM-based BSR with the Jeffreys' prior. Although there seems to be the possibility of further improvement of the accuracy by choosing the priors yielding more suitable degree of shrinkage for the estimates of SNP effects, it is generally difficult to construct such desirable prior for *σ*_*gl*_^2^.

An actual strategy to determine the optimal values of *p*, *v *and *S *would be to evaluate the accuracies obtained by varying the values of these hyperparameters in small steps over the suitable ranges, for example, 0 <*p *< 1, 0 <*v *< 5, 0 <*S *< 1. In genomic selection applied for the actual data, cross validation might be a method of choice for determining the suitable values of these hyperparameters. A number of replications in the estimation of a large number of SNP effects are necessarily required for finding the optimal values. When replicated estimations are required, the advantage of EM-based wBSR method over MCMC-based methods with respect to the computational time would be much more remarkable.

In [8], EM algorithm was applied for the shrinkage regression model of QTL mapping in the framework of generalized linear model, which included logistic model and probit model as well as normal linear model described in this study by choosing appropriate link functions, following [9]. For the EM algorithm applied to normal linear model described in [9], standardization of outcome variable by rescaling it to have mean 0 and standard deviation 0.5 was recommended. The influence of data transformation on the accuracies in the prediction of GBVs seems important as well as that of the prior settings for *gl *and *σ*_*gl*_^2^. These investigations would be described elsewhere.

In large-scale genotyping data used for genomic selection including tens of thousands SNP genotypes for thousands of individuals, a large number of SNP genotypes may still be missing. EM-algorithm allows the missing SNP genotypes to be inferred with posterior expectations of the indicator variables of genotypes given the information of the adjacent SNPs or pedigree information. A step for the inference of missing genotypes can also be included in our EM-based method of genomic selection. Although the inference of missing genotypes with EM-algorithm has been shown to be effective for increase in power of QTL detection, how prediction accuracy is affected by the inference of missing genotypes in genomic selection remains to be investigated. This topic should be addressed in the further study.

We developed a program implementing EM algorithm for estimating SNP effects, described here, in genomic selection and applied the program for the simulation study. The information of this program is provided below (see Availability and requirements).

## Conclusion

In this research, we described EM algorithm for a Bayesian method, BSR, that included effects of all SNPs in a regression model as covariates in genomic selection and was so far based on MCMC algorithm. Moreover, we devised a modified version of BSR method called wBSR by incorporating the weight assigned to each SNP according to the strength of its association with a trait, for which EM algorithm was also applicable. As results of simulation experiments, it was shown that the accuracy in predicting GBV by wBSR was improved in comparison with MCMC-based BSR. Although the accuracy of wBSR was inferior to SSVS, wBSR was regarded as a practical and cost-effective method taking great computing advantage over MCMC-based Bayesian methods into account.

## Availability and requirements

The source code of the program used in the simulation study was written with Fortran 77 and a Windows version of the executable program is available on the request to the first author (hayatk@affrc.go.jp). The sample input files and a brief manual of the program can be also provided.

## Authors' contributions

TH devised EM algorithm for Bayesian methods in genomic selection, developed a program for simulations and drafted the manuscript. HI assisted in developing a program and drafted the final manuscript. Both authors read and approved the final manuscript.
